# How can we improve knowledge and perceptions of menstruation? A mixed-methods research study

**DOI:** 10.1186/s12905-020-01007-4

**Published:** 2020-09-29

**Authors:** Gayoung Moon, Inkyung Kim, Habhin Kim, Suwan Choe, Soyeon Jeon, Jeonghun Cho, Sujeong Hong, Jisan Lee

**Affiliations:** 1 College of Nursing, Catholic University of Pusan, (46252) 117, College of Nursing, 57, Oryundae-ro, Geumjeong-gu, Busan, Republic of Korea; 2 College of Life & Health Sciences, Department of Nursing & The Research Institute for Basic Sciences, Hoseo University, (31499), RIC building, 20, Hoseo-ro 79 beon-gil, Baebang0eup, Asan-si, Chungcheongnamdo Republic of Korea

**Keywords:** Menstruation, Menstrual products, Young adult, Needs assessment, Sex education

## Abstract

**Background:**

Traditionally, menstrual education has consisted of lectures directed toward women. The objective of this study was to design an innovative menstrual education (ME) program that reflects the needs of both young women and men, and verify its effectiveness.

**Methods:**

A mixed-method design was used to determine the program needs and assess young adults’ knowledge and perceptions of menstruation and menstrual products. Focus group interviews were conducted with 14 young adults, and 150 young adults participated in an online survey. After developing the ME program, 10 young adults participated in a study to verify its effectiveness.

**Results:**

Interview results showed young adults wanted more information about menstrual products. The online survey revealed significant differences in knowledge based on participants’ general characteristics and experience; exposure to menstruation and menstrual products positively impacted knowledge and perception. In addition, the results indicated young adults wanted ME content access via mobile and in-person modalities, designed for both genders, drawing on menstrual experts’ knowledge. Based on these results, a multi-experimental menstrual education (MEME) program was designed and included: hands-on exposure to 60 menstrual products, product demonstrations with a female perineal model, a YouTube video created by the researchers, a true-or-false quiz, and question-and-answer sessions with menstrual experts.

**Conclusions:**

This study clarified the requirements of an innovative menstrual education program. It led to high satisfaction among participants, and improved knowledge and perceptions of menstruation and menstrual products. The online survey showed a correlation between the extent of received ME, and respondents’ perception of menstrual products. This implied that a MEME program could change perceptions when conducted systematically; by extension it could ameliorate menstruation challenges attributed to poverty. Future research could further verify the effectiveness of the MEME program, using a larger sample, and examine its suitability for incorporation into official ME curricula at universities and companies.

**Trial registration:**

This trial was registered in a Clinical Research Information Service in Korea linked with the World Health Organization’s International Clinical Trial Registry Platform (WHO’s ICTRP) (no. KCT0004715), Registered 07 Feb 2020.

## Background

Menstruation occurs when a woman’s body discharges blood and endometrial tissue through the vagina [1]. On average, women between the ages of 13 and 52 years experience a menstrual period lasting 3 to 5 days a month. Menstrual products are a necessity and are used regularly by women. However, the variety of products and their costs create challenges to menstrual hygiene management.

Poverty undermines menstrual hygiene management around the world. During one menstrual cycle, a woman may need 12 to 30 disposable pads [2–5]. A study in India found that only 40% of adolescent girls used commercially available pads to absorb menstrual blood, and more than half (60%) used old clothing due to price and accessibility issues [6]. In addition, the United Nations Children’s Fund published a study in which 10% adolescent girls in Western Kenya engaged in sexual intercourse to acquire money for menstrual pads [7, 8]. In Korea, due to the high price of disposable menstrual pads, it has been reported that girls use shoe insoles in lieu of menstrual products [9]. Moreover, Period.org, a not-for-profit corporation, noted 35 states in the United States have so-called “tampon taxes,” meaning that tampons are subject to value-added tax, unlike other necessities [8]. To resolve these problems, global perceptions of menstrual products need to change so they are recognized as necessities.

With an increasing number of issues and amount of interest in menstruation and menstrual products, many regulations and legislations are actively changing. At present, the U.S. Food and Drug Administration does not heavily regulate menstrual products such as menstrual pads and tampons, because they are classified as “medical devices,” which do not require ingredient labeling [10, 11]. However, in 2019, the New York City Council passed a senate bill requiring ingredient disclosure for menstrual products [12].

Menstruation and menstrual products are fundamental components of life, and both women and men should thoroughly understand them [13]. Therefore, it is necessary to examine and improve the current knowledge and perceptions of women and men on this topic. One method is through education.

In Korea, public school students receive sex education, but the curriculum does not comprehensively address menstruation or menstrual products and most students are dissatisfied with its content (Woo MJ: Godeunghagsaeng-ui seongjisig mich taedowa seong-gyoyug yogudo-e gwanhan yeongu [study on sexual knowledge of high school students and their demands for sex education], Unpublished). Jun and Lee [14] revealed the negative impact of unilateral sex education (via lecture) on students’ attitudes toward menstruation. Another study has shown a tendency to provide more intensive menstrual education (ME) for women than men, which leads to misinformation among men [15]. Furthermore, this misinformation perpetuates broader misunderstandings about reproductive health, and opportunities to correct this seldom arise as information dissemination is limited at home and in school [16]. These circumstances imply the need for innovative ME programs. This study aimed to design an innovative ME program that reflects the needs of both young women and men and to verify its effectiveness.

## Methods

### Research design

The study used a mixed-methods design comprising 3 elements: a focus group interview (FGI), an online survey, and an intervention (ME).

**Fig. 1:**
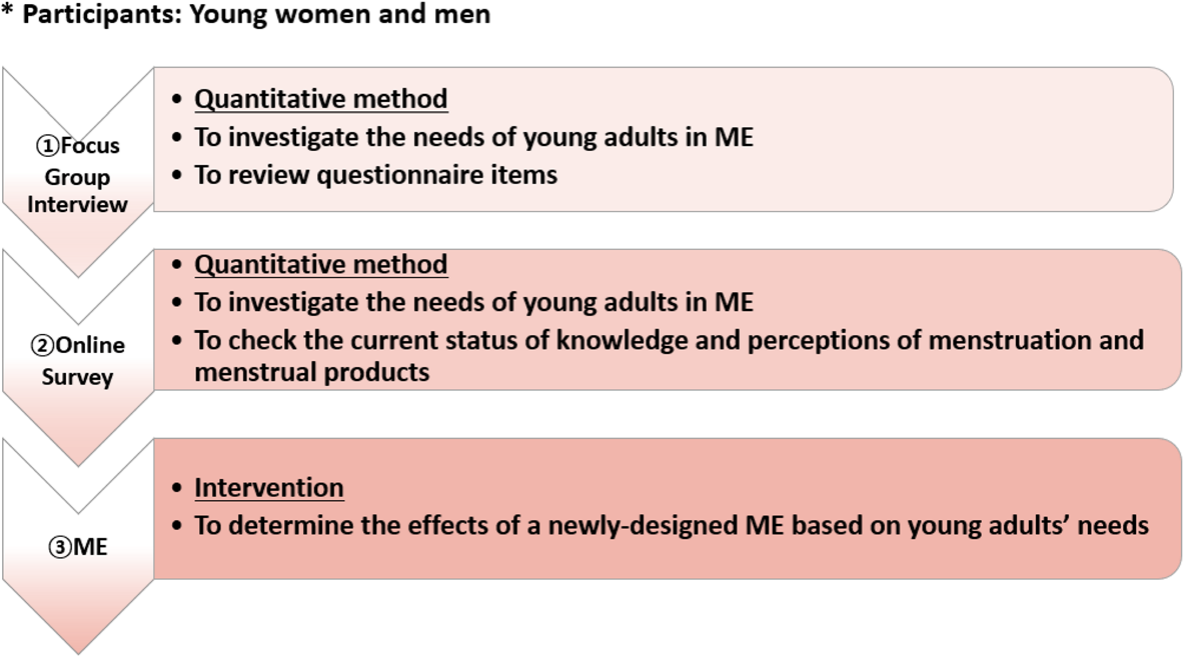
The design of this study and method. *Note.* ME = menstruation education

The FGIs investigated the needs of young adults related to ME. The interviews were semi-structured and based on a questionnaire. The online survey ascertained young adults’ current knowledge and perceptions of menstruation and menstrual products and ME content needs. The effects of a newly designed ME program were determined. Figure 1 shows the current research design in detail.

### Participants and data collection

Eligible participants were women and men aged 20 years or more, who had graduated from high school and were unmarried. These criteria were applied to ensure a population with similar ME experience and because marriage and pregnancy could affect women’s and men’s experience with menstruation and menstrual products. Recruitment was conducted via snowball sampling using the social network platforms: KakaoTalk and Facebook. The online survey was administered using Survey Monkey. To obtain information concerning the knowledge, perceptions, and needs of both women and men, we included equal proportions of women and men in the FGIs and the online surveys. About 90% FGIs on health-seeking behaviors used three to six group and focus group sample sizes could differ from many of the “rule-of-thumb” recommendations. In this study, FGIs was conducted with 7 groups of 2 participants each [17]. G*power 3.1.9 was used to calculate the sample size for online survey with an effect size of .3, significance of .05, and power of .95 [18]. In total, 138 participants were required. Accordingly, a sample of 150 participants was thought best (considering dropout rate). Data were collected from December 2018 to January 2019.

### Measures

A questionnaire was used in FGI and online survey, and intervention (ME). After FGI, some of the items that interviewees reported as being difficult or confusing were modified. For example, the item about disposable menstrual products was changed to “disposable menstrual pad,” and the question whether participants had ever “encountered” menstrual items was modified to whether they had actually “seen” them.

The questionnaire contained 65 items. Most items had been extracted, corrected, and supplemented from previous studies and some items needed in this study were developed by the authors. Nine items were about general characteristics including sex, age, level of university education, university major, and various factors that could influence knowledge and perceptions related to menstruation and menstrual products, such as the presence of female siblings, dating experience, and real-life experience of menstruation or menstrual products. Four items were about ME experiences: four items were used to examine menstrual knowledge acquisition pathways and age of receiving and contents of sex education (Jeong S: 20 dae namnyeoui seong-gyeongheom mich seong-gyoyug siltae Josa [a survey on state of sexual experience and sexual education of 20s men and women], Unpublished).

Menstruation knowledge was evaluated with 13 items (Yoon J: Chodeunghaggyoui wolgyeong-gyoyughyeonhwang-e ttaleun yeohagsaengdeul-ui wolgyeongjisiggwa taedo [menstrual knowledge and attitude of female students according to the current status of menstrual education in elementary school], Unpublished), (Lee Y: CAIleul hwal-yonghan hugi haglyeong-gi adong-ui mongjeong-Gwa wolgyeong gyoyug peulogeulaem gaebal mich hyogwa [Development and effects of nocturnal emission and menstruation education program using cai for Korean elementary school children], Unpublished), (Jung M: Yeogosaeng-ui wolgyeongjisig, taedo, jeungsang mich daecheoe gwanhan yeongu [A study on the menstrual knowledge, attitudes, symptoms and coping of the high school girls], Unpublished). We scored these items by assigning one point to each correct answer, with a higher score (out of 13) indicating greater menstruation knowledge. The Cronbach’s alpha of this section was .794. Knowledge of menstrual products was measured using 14 items developed with reference to information from the Korean Food and Drug Administration [19] and Korea Cotton Sanitary Brands (Gnaran: Wrong information and effective washing methods; unpublished). Correct answers were scored one point, with a higher score (out of a possible 14) indicating a higher level of knowledge of menstrual products. The Cronbach’s alpha of this section was .845.

Participants’ perceptions of menstruation and menstrual products were measured using 16 items. Eleven items were from previous studies and 3 were developed for this study pertaining to insertable menstrual products and menstrual products other than disposable pads [20, 21]. For this section, items were rated on a five-point Likert scale (5 = completely agree; 1 = completely disagree). The total score ranged from 16 to 80, with a higher score indicating a more positive perception of menstruation and menstrual products. The reliability of this section was .729. Five items about their experiences and needs concerning ME and 4 items about usage of menstrual products (only for women participants) were developed by the authors. The IRB-approved questionnaire file used in this study show these in more detail (see Supplementary file 1).

### Data analysis

The collected data were analyzed with SPSS Statistics 25. The data were analyzed using frequency statistics, means, standard deviations, an independent t-test, Pearson’s correlation coefficient, the Wilcoxon signed-rank test, and Cronbach’s alpha.

## Results

### Focus group interviews

Fourteen unmarried young adults (7 women and 7 men) were included in the FGIs in groups of two regardless of sex.

#### ME needs

Regarding the limitations of traditional sex education, the FGI participants stated, “*Nothing in particular about the information I had previously received through such education programs was useful to me.*” They also noted that *“I had acquired little knowledge in these programs.”* Female participants were asked about their use of menstrual products; all of them said that *“I have used only disposable menstrual pads because these were commonly used and familiar, and I am afraid of using insertable products.”* Elaborating upon this, they said, “*We need education related to the variety of menstrual products, as our knowledge is centered mostly on disposable menstrual pads”* and *“I had no opportunity to see or touch the various menstrual products. It would be good if I could get a lot of information about the various types of menstrual products and the advantages, disadvantages,and side-effects of each product.*” Furthermore, the participants preferred education using smartphones (e.g., through social networking services or apps) (*n* = 6, 42.9%), because of the accessibility, convenience, diversity, and affordability of this mode, followed by an in-person lecture (*n* = 5, 35.7%) in which an expert provides professional knowledge and offers opportunities for communication (e.g., question-and-answer [Q&A] sessions).

#### Knowledge and perceptions of menstruation and menstrual products

Regarding knowledge and perceptions of menstruation and menstrual products, the mean score for menstruation knowledge was 9.43 out of 13 (10.0, 76.9% for women and 9.0, 68.2% for men) and that for menstrual product knowledge was 8.36 out of 14 (9.6, 68.4% for women and 7.1, 51.0% for men). The mean score for perceptions of menstruation was 5.02 out of 7 (5.1, 72.9% for women and 4.9, 70.6% for men) and that for perceptions of menstrual products was 4.64 out of 7 (4.7, 67.0% for women and 4.6, 65.7% for men).

Interestingly, there were no large differences between the number of correct answers among women and that among men regarding menstruation and menstrual product perceptions. When answering the interview question related to menstruation and menstrual product perceptions, 5 men (71.4%) reported that *“Dating had influenced my menstrual perception,”* and that *“I gained awareness of the actual symptoms of menstruation by observing my partners before and during menstruation.”* Additionally, when asked about the situation of purchasing menstrual products, 7 women and 4 men participants (78.5%) answered “*I am not conscious of others when purchasing menstrual products,*” and “*Because menstruation is a natural phenomenon, it is not necessary to hide it.*”

Three women and 4 men responded that they found these items difficult to answer and confusing, whereas 2 women and 1 man found answering them easy. Based on participants’ responses, the questionnaire was revised to include words that were clearer and easier to understand (e.g., “disposable menstrual product” was changed to “disposable menstrual product (pad)”).

### Online survey

#### General characteristics

A total of 150 participants completed the survey. As noted in Table 1, the participants were similar in terms of sex, university major, and presence of a sister. Furthermore, for the item evaluating experience of seeing menstrual products first-hand, disposable menstrual pads (*n* = 136; 90.7%) were reported as the most seen and 10 men (6.7%) had never seen such products.

**Table 1:** General Characteristics of Participants of the Online Survey (*N* = 150)

	Classification	n (%)	M (±SD)
Gender	Women	75 (50.0)	
	Men	75 (50.0)	
Major	Health-related department	71 (47.3)	
	Non-health-related department	79 (52.7)	
Year of university	1st year	14 (9.3)	
	2nd year	29 (19.3)	
	3rd year	40 (26.7)	
	4th year	67 (44.7)	
Age (years)			23.18 (±1.850)
Presence of sister	Yes	74 (49.3)	
	No	76 (50.7)	
	Women - Yes	39 (52.0)	
	Women - No	36 (48.0)	
	Men - Yes	35 (46.7)	
	Men – No	40 (53.3)	
Dating experience	Yes	125 (83.3)	
	No	25 (16.7)	
	Women - Yes	59 (78.7)	
	Women - No	16 (21.3)	
	Men - Yes	66 (88.0)	
	Men – No	9 (12.0)	
Had experienced or seen menstrual products first-hand (Duplicate selection)	Disposable menstrual pad	136 (90.7)	
	Reusable menstrual pad (Cotton menstrual pad, Menstrual panties)	55 (36.7)	
	Tampon	59 (39.3)	
	Menstrual cup	15 (10.0)	
	Panty liner	74 (49.3)	
	Never seen any	11 (7.4)	

### Usage of menstrual products

All the female participants had used disposable menstrual pads. Panty liners (*n* = 43, 57.3%), tampons (*n* = 17, 22.7%), reusable menstrual pads (*n* = 14, 18.7%), and menstrual cups (*n* = 1, 1.3%) were used with disposable menstrual pads. In terms of criteria for their selection of menstrual products, 44 women (58.7%) prioritized convenience, followed by safety (i.e., not an insertable type) (*n* = 14, 18.7%), functionality (*n* = 10, 13.3%), and affordability (*n* = 6, 8%). Interestingly, disposable menstrual pads were perceived to be the most inconvenient (*n* = 43, 57.3%). The main reason for the discomfort of disposable menstrual pads was their association with dermatitis and vaginitis (*n* = 16, 37.2%), followed by anxiety about issues such as menstrual blood leaking out of the pad (*n* = 12, 27.9%), the high cost and the smell of menstrual blood (*n* = 5, 11.6% for each), and concerns about potentially hazardous materials in the pad (*n* = 2, 4.7%).

### ME experience

Regarding previous ME experience, 90 participants (60%) had previous experience of menstruation education and 43 (28.7%) had previous experience of menstrual product education. They received ME in kindergarten or a children’s home (*n* = 21, 14%), elementary school (*n* = 109, 72.7%), middle school (*n* = 138, 92%), high school (*n* = 130, 86.7%), and university (*n* = 46, 30.7%; statistics include duplicate responses). On average, participants underwent 2.96 ± 1.03 instances of ME. Participants’ preferred pathways to receive information about menstruation and menstrual products were as follows: in-person lectures (*n* = 47, 31.3%), smartphones (e.g., social networking sites and apps; *n* = 41, 27.3%), and computers (e.g., portal services; *n* = 29, 19.3%).

### General characteristics and knowledge and perceptions of menstruation and menstrual products

The mean scores of menstruation and menstrual product knowledge among women were significantly higher than those among men. Moreover, there was a statistically significant difference between participants’ majors. Menstruation and menstrual product knowledge increased as participants’ year of university increased. Interestingly, there were no significant correlations between age and menstruation or menstrual product knowledge (Table 2).

**Table 2:** Menstruation and Menstrual Product Knowledge and Perceptions according to General Characteristics (*N* = 150)

		Menstruation Knowledge			Menstrual Product Knowledge			Menstruation Perceptions			Menstrual Product Perceptions		
	Classification	*M* (*SD*)	*t*	*p*	*M* (*SD*)	*t*	*p*	*M* (*SD*)	*t*	*p*	*M* (*SD*)	*t*	*p*
Gender	Men (*n* = 75)	8.11 (3.32)	−5.770	< .01*	5.04 (3.36)	−9.457	< .01*	26.60 (3.53)	−0.383	.702	8.11 (3.31)	−2.486	.014*
	Women (*n* = 75)	10.59 (1.69)			9.53 (2.43)			26.81 (3.29)			10.59 (1.69)		
University major	Health-related (*n* = 71)	10.21 (2.46)	3.593	< .01*	8.30 (3.42)	3.193	< .01*	10.21 (2.46)	3.593	< .01*	27.06 (3.17)	1.203	.231
	Non-health-related (*n* = 79)	8.56 (3.06)			6.42 (3.75)			8.57 (3.06)			26.39 (3.59)		
University year	1st year (*n* = 14)	7.36 (3.08)	*F* = 5.799	< .01*	4.79 (3.29)	*F* = 7.047	< .01*	26.85 (4.28)	*F* = 0.113	.953	29.50 (4.91)	*F* = 2.653	.051
	2nd year (*n* = 29)	8.48 (2.95)			5.59 (3.75)			26.38 (2.65)			27.86 (4.72)		
	3rd year (*n* = 40)	9.15 (3.26)			7.60 (3.09)			26.75 (3.33)			29.65 (4.20)		
	4th year (*n* = 67)	10.25 (2.28)			8.40 (3.66)			26.79 (3.59)			30.75 (4.79)		
Presence of sister	Yes (*n* = 74)	9.32 (3.07)	−0.093	.926	7.26 (3.76)	−0.162	.871	26.89 (3.69)	−1.291	0.199	28.32 (3.71)	−2.682	< .01*
	No (*n* = 76)	9.37 (2.75)			7.36 (3.68)			27.70 (3.94)			30.02 (4.05)		
Dating experience	Yes (*n* = 125)	9.42 (2.90)	0.653	.515	7.48 (3.65)	1.284	.201	26.91 (3.45)	1.663	0.098	29.74 (4.72)	−0.029	0.835
	No (*n* = 25)	9.00 (2.97)			6.44 (3.93)			25.68 (2.98)			29.96 (4.77)		
	*M* (*SD*)		*r*	*p*		*r*	*p*		*r*	*p*		*r*	*p*
Age	23.18 (1.85)		0.083	.311		−0.036	.666		−0.035	0.668		0.008	0.926
Number of menstrual products seen	2.25 (1.37)		0.356	< .01*		0.549	< .01*		0.126	0.126		0.348	< .01*

* *p* < .05

Participants’ perceptions of menstruation and menstrual products were statistically significant according to sex, university major, year of university, and presence of a sister. Additionally, the number of menstrual products seen first-hand had a statistically significant effect on menstruation and menstrual product knowledge and perceptions (Table 2). In other words, those who had seen various menstrual products had significantly better menstrual product knowledge and perceptions than did those who had not seen them.

### ME experience and knowledge and perceptions of menstruation and menstrual products

Participants who had received ME or menstrual product education had more knowledge and more positive perceptions than did those who had not, but this difference was not statistically significant. Interestingly, the mean scores for menstruation and menstrual product knowledge were significantly higher among those who had received ME or menstrual product education.

To determine the effect of the ME, correlation analysis was conducted. The number of ME received was related to menstruation and menstrual product knowledge and perceptions (Table 3).

**Table 3:** Differences in Knowledge of Menstruation and Menstrual Products: Experience of ME (*N* = 150)

		Menstruation knowledge			Menstrual product knowledge			Menstruation perceptions			Menstrual product perceptions		
	Classification	M (SD)	*t*	*p*	M (SD)	*t*	*p*	M (SD)	*t*	*p*	M (SD)	*t*	*p*
ME experience													
	Yes (*n* = 90)	10.18 (1.80)	4.034	< .01*	8.09 (3.19)	3.106	< .01*	27.01 (3.15)	1.347	.180	30.08 (4.89)	0.938	.345
	No (*n* = 60)	8.10 (3.71)			6.13 (4.12)			26.25 (3.73)			29.23 (4.44)		
Menstrual product education experience													
	Yes (*n* = 43)	10.07 (1.93)	2.383	.019*	8.47 (2.93)	2.780	< .01*	27.41 (3.44)	1.635	.104	30.91 (5.06)	1.873	.063
	No (*n* = 107)	9.06 (3.17)			6.84 (3.89)			26.42 (3.36)			29.33 (4.51)		
		M (SD)	*r*	*p*		*r*	*p*		*r*	*p*		*r*	*p*
Amount of ME received													
		2.96 (1.03)	0.193	.018*		0.065	.430		−0.038	0.645		0.166	.043*

*ME* menstrual education

*p < .05

A statistically significant correlation was found between the knowledge of and the perceptions of menstruation and menstrual products (Table 4).

**Table 4:** Correlation between Menstruation and Menstrual Product Knowledge and Perceptions (*N* = 150)

	Menstruation knowledge	Menstrual product knowledge	Menstruation perceptions	Menstrual product perceptions
Menstruation knowledge	1			
Menstrual product knowledge	0.627*	1		
Menstruation perceptions	0.095	0.137	1	
Menstrual product perceptions	0.281*	0.347*	0.517*	1

*p < .05

### ME needs

The largest proportion of participants had needs related to education contents on the use of menstrual products (*n* = 96, 64%), followed by the various types of menstrual products and their advantages and disadvantages (*n* = 89, 59.3%). Participants also needed information on the management of menstruation (*n* = 89, 59.3%), side effects of menstrual products (*n* = 72, 48%), and physiological mechanism of menstruation (*n* = 53, 35.3%; statistics include duplicate responses).

In terms of the method of ME, participants expressed the desire to see menstrual products first-hand (*n* = 80, 53.3%) or audiovisually (e.g., a YouTube video; *n* = 79, 52.6%). Other preferred methods were slideshow presentations (*n* = 52, 34.7%), pamphlets (*n* = 43, 28.7%), face-to-face discussions (*n* = 21, 14%), and in-person lecture by menstruation experts (*n* = 20, 13.3%; statistics include duplicate responses).

### MEME

Finally, the two-hour ME program was designed based on the needs of young adults: a multi-experiential menstruation education (MEME). The MEME program was conducted by providing in-person lectures using a YouTube video. This approach was implemented to allow participants to view the lecture content on their smartphones at any time after the lecture. The features of the MEME program were as follows:

A. Watching a YouTube video that we created: The YouTube video focused on the mechanism of menstruation and the types, methods of use, and side effects of menstrual products
B. Mini quiz about video contents
C. Hands-on experience with 60 menstrual products
D. Simulation on the use of these products using a female perineal model
E. Q&A sessions with menstruation experts.

**Fig. 2:**
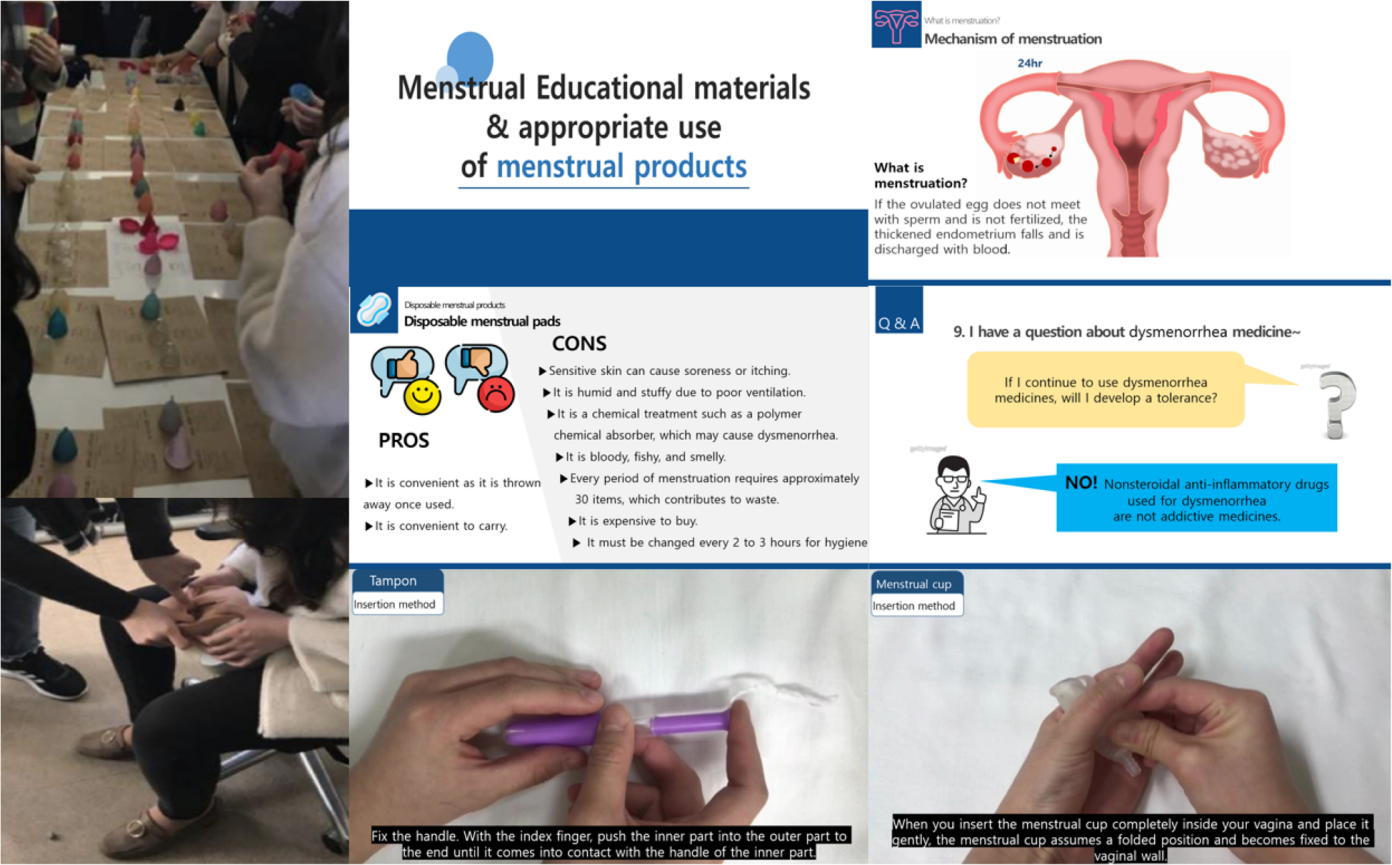
Educational contents of MEME in the form of a slideshow presentation and video (developed ourselves). *Note 1.* MEME = multi-experiential menstruation education

Figure 2 shows details of the MEME program. The video was uploaded to YouTube to enable other young adults to access them after the MEME program had been completed (https://www.youtube.com/watch: This link will be made public after review).

### General characteristics

This experiment included 10 participants (8 women and 2 men). All participants were university students. Their mean (SD) age was 22.90 (±0.88) years old. Their university majors were in health-related departments (*n* = 6, 60%) or non-health-related departments (*n* = 4, 40%). Six participants had a sister (60%) and 4 did not (40%); 7 participants had dating experience (70%) and 3 (30%) did not. Regarding their experience of seeing menstrual products first-hand, all the participants had seen disposable menstrual pads (100%), 3 had seen reusable menstrual pads (30%), 4 had seen tampons (40%), 3 had seen menstrual cups (30%), and 7 had seen panty liners (70%).

### Effect on the MEME program

After the MEME program, participants had significantly higher mean scores for knowledge of menstruation and menstrual products compared to pre-intervention. Perceptions of menstruation and menstrual products were also higher after the MEME program, although the differences were not significant (Table 5).

**Table 5:** Changes in Menstruation and Menstrual Product Knowledge and Perceptions after the MEME program (*N* = 10)

Classification	*M* (*SD*)		*Z*	*p*
	Before MEME	After MEME		
Menstruation knowledge	8.70 (2.63)	11.30 (1.49)	−2.820	< .01*
Menstrual product knowledge	8.70 (2.95)	13.00 (1.05)	−2.812	< .01*
Menstruation perceptions	9.70 (4.24)	43.20 (4.42)	−1.588	.112
Menstrual product perceptions	18.10 (2.77)	19.60 (1.27)	−1.479	.139

*MEME* multi-experiential menstruation education; * *p* < .05

In the Q&A sessions with the menstruation expert, the most frequently asked questions were related to the management and side effects of menstrual cups (*n* = 4, 40% each), followed by the selection (*n* = 3, 30%) and purchasing of a menstrual cup (*n* = 1, 10%; statistics include duplicate responses).

The participants stated that *“The YouTube video and Q&A sessions with menstruation experts were very helpful,” “Previous education was unengaging with studying theory using PowerPoint Presentation. Today, I am very pleased to be able to touch the various real menstrual items after a detailed video and receive a demonstration with a model,”* and *“Education in high school was a group education so I could not ask any questions personally. But as today’s education had a small number of people, I was able to ask the experts questions.*” Specifically, two men answered, *“I learned more details of menstruation and if these kinds of ME are held in the future, I would like to attend again.”*

## Discussion

In the current study, the response from our participants regarding their need for ME and first-hand experience of various menstruation products overwhelmingly suggest that even women who use only one type of menstrual product may feel limited and have a high interest in and demand for other menstrual products. The usage rate of various menstrual products in South Korea—especially menstrual cups (1.3%)—was remarkably low compared to other countries. In this study, all female participants reported using disposable menstrual pads. However, a study in the United States found that 31% women used tampons, 18% used disposable menstrual pads, and 51% used both products together [22]. More recently, in France, only 21% women were using disposable menstrual pads alone, while 9% were using menstrual cups; moreover, the different menstrual products were often used together, especially tampons, disposable menstrual pads, and panty liners [23]. These findings highlight the fact that Korean women appear to show a bias regarding the use of disposable menstrual pads. The persistence of this bias is interesting, given that more than half of the female participants in our study said disposable menstrual pads were uncomfortable to use. This result echoes the findings of Kim and Choi regarding women’s complicated relationship with disposable menstrual pads; while these pads are frequently used, consumers tend to be dissatisfied with the product [24]. The researchers attributed the persistent use to the ease of purchase and lack of suitable alternatives. That is, education that allows participants to touch, demonstrate, and discuss with an expert, such as the MEME program, could lower the barrier to finding alternatives for menstrual supplies that women currently use.

The mean scores for knowledge of menstruation and menstrual product were significantly higher among women, which is consistent with the findings of similar studies with college students [9, 25]. Additionally, knowledge scores were higher among students majoring in a health-related department and among those at a higher university level. Interestingly, there were no significant correlations between age and menstruation or menstrual product knowledge. However, this is not directly related to age. Rather, it means that knowledge and perception about menstruation and menstrual products do not simply increase with age, but with education and experience in society. Furthermore, the knowledge and perception were higher among those who had varied experience of menstruation products, suggesting that exposure to menstruation and menstrual products enhances their knowledge and perception. This implies an increased need for experiential education on menstruation and menstrual products like MEME, consistent with the findings of other studies [26].

Though women’s perceptions tended to be more positive, both men and women had positive perceptions of menstruation and menstrual products. Kang obtained similar results [9]. However, Hwang found significantly more negative attitudes in women than men [25]. This contradiction indicates the need for further research on menstrual perception based on sex. Moreover, given the low usage rates of insertable menstrual products in previous research and the current study, it may be necessary to explain in further studies why individuals perceive insertable menstrual products in a positive way but as unusable.

We found a significant positive correlation between knowledge and perception of menstruation and menstrual products, suggesting that greater knowledge facilitates less prejudicial perception. What are the clinical implications of these findings? In previous study, the effects of endometriosis, a characteristic symptom of menstrual pain, on psychological, social factors and quality of life have already been identified [27, 28]. In a future study, it could be possible to further study the effect of knowledge and perception of menstruation and menstrual products on the psychological, social factors and behavior changes of women experiencing dysmenorrhea. If these studies are conducted, ME could be used to determine the impact on women’s health management and if so, MEME could be used in real clinical settings.

The MEME was found to facilitate an increase in both knowledge and perception of menstruation and menstrual products. Changes in only knowledge was statistically significant. Participants noted that the unconventional education style was interesting and informative, that their perception of menstrual products other than disposable menstrual pads had positively changed, and that the program had provided them the opportunity to correct their misunderstandings. Moreover, the use of a female perineal model with various menstrual products and a Q&A with an expert helped to resolve their personal questions, unlike previous education in school. These findings are similar to the results of Yoon, showing that non-traditional forms of education, such as demonstrations or debates, were more effective in this context. In our study, the MEME facilitated positive growth in both knowledge and perception, suggesting that this might also be a useful strategy in sex education.

## Conclusion

This study aimed to design an ME based on young adults’ needs that differs from conventional sex/menstruation education and verify its effectiveness. The strength of this study was that it utilized various new research methods. To understand young adults’ needs, we conducted FGIs and an online survey. We then created ME content available on mobile and in-person education for both sexes with menstruation experts and multi-experimental methods. Finally, we developed a MEME program, which consisted of a YouTube video that we created, a mini quiz, hands-on experience with 60 menstrual products, simulations of the use of these products using a female perineal model, and Q&A sessions with menstruation experts.

After implementation, it was found that the program not only led to high satisfaction among participants, but also helped to improve their knowledge and perception of menstruation and menstrual products. Additionally, the online survey results showed that the number of ME received was correlated with respondents’ perception of menstruation and menstrual products and menstruation and menstrual knowledge. The limitation of this study was that MEME was validated in a small group of asymmetric genders. This study implies that reiterative application of MEME could change perceptions and, by extension, could clarify and address the causes of poverty-related problems regarding menstrual products. To achieve the latter, MEME should be further verified, using a larger sample, which would also enable its use in the field, for example at universities and companies.

## Supplementary information

**Additional file 1.**

## Data Availability

The data that support the findings of this study are available from the corresponding author upon reasonable request.

## Abbreviations

FGI: Focus group interview
ME: Menstruation Education
MEME: Multi-Experiential Menstruation Education
Q&A: Question-and-answer
