# Supplementary material for: How can we improve knowledge and perceptions of menstruation? A mixed-methods research study
Source: BMC Womens Health. 2020 Sep 29;20:214. doi: 10.1186/s12905-020-01007-4 (PMC7526121; doi:10.1186/s12905-020-01007-4)
Supplement: Supplementary file 1 — Additional file 1. [file 12905_2020_1007_MOESM1_ESM.docx]

**Questionnaire**

**1. General Characteristics**

01. What is your gender?

① Female ② Male

02. What is your age? ( )

03. Where is your current university located?

① Busan ② Seoul and metropolitan area (Gyeonggi Province) ③ Other

04. What is your current year of study in the university?

① 1^st^ year ② 2^nd^ year ③ 3^rd^ year ④ 4^th^ year

05. What is your major?

① Public health ② Humanities ③ Sciences ④ Engineering ⑤ Arts and Physical Ed. ⑥ Other ( )

06. What is your subjective perception of your economic level?

① Upper ② Upper-middle ③ Middle ④ Lower-middle ⑤ Lower

07. Do you have a sister?

① Yes ② No

08. Do you have any dating experience?

① Yes ② No

09. Which of the following menstrual products have you seen or had first-hand experience with? (Duplicate selection)

① Disposable menstrual pads ② Reusable menstrual pads (Cotton menstrual pad, Menstrual panties)

③ Tampons ④ Menstrual cups ⑤ Panty liners ⑥ Never seen any ⑦ Other ( )

**2. Menstruation Education Experience**

01. To the best of your recollection, at what stages have you had sex education in your school? (Duplicate selection)

① Children’s home ② Elementary school ③ Middle school ④ High school ⑤ University and above ⑥ Never

02. Please select all topics of sex education that you have previously had. (Duplicate selection)

① Gender equality ② Sexual desire control (masturbation) ③ Homosexuality

④ Puberty and secondary sex characteristics ⑤ Pregnancy & birth ⑥ Contraception
⑦ Prevention of sexually transmitted diseases (STDs) / AIDS ⑧ (Induced) Abortion
⑨ Prevention of sexual assault (crime) ⑩ Structure and function of male and female genitals
⑪ Menstruation ⑫ Menstrual products ⑬ Responding to pornography ⑭ Other ( 　 　)

03. From where do you acquire most of your knowledge regarding sex?

① Sex education classes ② TV ③ Computer (Internet, Web portal)

④ Smartphone (SNS, APP) ⑤ Sex education books ⑥ Printouts (magazines, books, etc.)

⑦ Friends ⑧ Parents ⑨ Other ( )

04. What is your preferred method for obtaining information on sex?

① Classes/lectures on sex education ② TV ③ Computer (Internet, Web portal)

④ Smartphone (SNS, APP) ⑤ Sex education books ⑥ Printouts (magazines, books, etc.)

⑦ Friends ⑧ Parents ⑨ Other ( )

**3. Knowledge of Menstruation**

| Question | Category | Yes | No | Do not know |
| --- | --- | --- | --- | --- |
| 1 | Menstrual blood consists entirely of blood. |  |  |  |
| 2 | Menstruation is a process that involves bleeding when the endometrium detaches due to female hormones. |  |  |  |
| 3 | Although the typical menstruation period is 4–6 days, a menstruation period of 2–8 days is also considered normal. |  |  |  |
| 4 | The amount of menstrual discharge is consistent throughout the period (from the start date to the end date). |  |  |  |
| 5 | The menstrual cycle is 25–36 days in general, although it varies with each individual. |  |  |  |
| 6 | Premenstrual syndrome (PMS) involving acute pain or emotional changes may occur, often immediately before menstruation (thus indicating that menstruation will soon commence). |  |  |  |
| 7 | During a menstruation period, there is no need to change one’s underwear as menstrual pads are worn. |  |  |  |
| 8 | All females experience menstrual cramps. |  |  |  |
| 9 | One should take pain medications every time menstrual cramps are experienced. |  |  |  |
| 10 | Menstrual cramps are a sign of poor health. |  |  |  |
| 11 | Vaginal cleansing during menstruation helps prevent vaginitis (vaginal inflammation). |  |  |  |
| 12 | Taking a shower is okay during menstruation, but one should avoid taking a full bath. |  |  |  |
| 13 | Consuming food products that are rich in calcium can help alleviate menstrual cramp symptoms. |  |  |  |

**4. Knowledge of Menstrual Product**

| Question | Category | Yes | No | Do not know |
| --- | --- | --- | --- | --- |
| 1 | There are different sizes of disposable menstrual pads (large, medium, small) for different physiques. |  |  |  |
| 2 | In general, one disposable menstrual pad is used per day. |  |  |  |
| 3 | Disposable menstrual pads should be wrapped and discarded so that others cannot see. |  |  |  |
| 4 | Used cotton menstrual pads should not be washed using a washer. |  |  |  |
| 5 | Menstrual panties (non-leaking panties) should be used after attaching a disposable menstrual pad. |  |  |  |
| 6 | Tampons should be used immediately after opening. |  |  |  |
| 7 | Tampons should not be used for longer than 8 hours. |  |  |  |
| 8 | When inserting a tampon, the handle should not enter the vagina. |  |  |  |
| 9 | After inserting a tampon, the string should be left as it is. |  |  |  |
| 10 | For menstrual cups, a larger size is better. |  |  |  |
| 11 | A menstrual cup can be used continuously if sanitized after use. |  |  |  |
| 12 | A menstrual cup should be sanitized with alcohol before use. |  |  |  |
| 13 | A menstrual cup can be used without previous sexual experience. |  |  |  |
| 14 | If properly sanitized, a menstrual cup can be shared with others. |  |  |  |

**5. Perception of Menstruation and Menstrual Products**

| Question | Category | Strongly disagree | Disagree | Average | Agree | Strongly agree |
| --- | --- | --- | --- | --- | --- | --- |
| 1 | Menstruation is a symbol of feminine beauty. |  |  |  |  |  |
| 2 | I wish that menstruation did not exist. |  |  |  |  |  |
| 3 | I feel embarrassed / awkward when seeing advertisements for menstrual products (i.e., menstrual pads, pain medications for menstrual cramps) on TV or in magazines. |  |  |  |  |  |
| 4 | Menstrual products should be hidden since they are secretive, feminine products. |  |  |  |  |  |
| 5 | One should not talk about menstruation in public. |  |  |  |  |  |
| 6 | I feel embarrassed talking about menstruation with a person of the opposite sex. |  |  |  |  |  |
| 7 | I become self-conscious when purchasing menstrual products. |  |  |  |  |  |
| 8 | Females cannot demonstrate the same competence as males due to menstruation. |  |  |  |  |  |
| 9 | It seems like female students who complain of menstrual cramps are simply exaggerating. |  |  |  |  |  |
| 10 | Female students are easily annoyed and depressed during menstruation. |  |  |  |  |  |
| 11 | Menstrual leave (or absence due to menstruation) is necessary. |  |  |  |  |  |
| 12 | I feel that the use of insert-type menstrual products (i.e., tampons, menstrual cups) may cause a loss of virginity. |  |  |  |  |  |
| 13 | I feel that insert-type menstrual products (i.e., tampons, menstrual cups) are difficult to use. |  |  |  |  |  |
| 14 | I feel that the use of insert-type menstrual products (i.e., tampons, menstrual cups) can result in reduced sexual intercourse satisfaction. |  |  |  |  |  |
| 15 | Reusable (cotton) menstrual pads are insanitary. |  |  |  |  |  |
| 16 | Aside from disposable menstrual pads, all menstrual products are expensive. |  |  |  |  |  |

**6. Menstrual Education Needs**

Responses to the following questions will be reflected in the production of new educational materials. Please read carefully and select the most appropriate answer(s).

01. What content would you like to see in educational materials on menstruation and menstrual products? (Duplicate selection)

① Mechanism of menstruation (physical changes) ② Types and pros/cons of menstrual products

③ How to use menstrual products ④ How to handle menstruation ⑤ Adverse effects of menstrual products

⑥ Other ( ）

02. How would you like to receive education on menstruation and menstrual products?

① Pamphlets ② PowerPoint-based education ③ Video material

④ Hands-on experience of menstrual products ⑤ Discussion (Group discussion) ⑥ 1:1 interview ⑦ Other ()

03. Would you be interested in newly produced educational materials on menstruation/menstrual products

① Yes ② No

04. Would you be interested in participating in offline educational programs on menstruation/menstrual products?

① Yes ② No

05. Please elaborate on the unsatisfactory aspects of traditional sex education.

- Type (form) of education:
- Unsatisfactory aspects:

**7. Usage of Menstrual Products (for women)**

01. Which of the following menstrual products are you currently using? (Duplicate selection)대로 나열해

① Disposable menstrual pads ② Reusable menstrual pads (Cotton menstrual pad, Menstrual panties)

③ Tampons ④ Menstrual cups ⑤ Panty liners ⑥ None ⑦ Other ( )

02. Which of the following menstrual products have you had experience with? (Duplicate selection)

① Disposable menstrual pads ② Reusable menstrual pads (Cotton menstrual pad, Menstrual panties)

③ Tampons ④ Menstrual cups ⑤ Panty liners ⑥ None ⑦ Other ( )
03. What are your key selection criteria for choosing menstrual products?

① Convenience ② Safety ③ Functionality ④ Economic value ⑤ Other ( )

04. Please elaborate on the unsatisfactory aspects of menstrual products.

- Type of menstrual product:
- Unsatisfactory aspects:

As a token of our gratitude, we would like to give all participants a small gift. For this, please write your phone number below so we can contact you.

( )

We thank you for your time and sincere responses to the questionnaire.
